# Hydrogen-Rich Saline Protects against Ischemia/Reperfusion Injury in Grafts after Pancreas Transplantations by Reducing Oxidative Stress in Rats

**DOI:** 10.1155/2015/281985

**Published:** 2015-03-22

**Authors:** Zhu-Lin Luo, Long Cheng, Jian-Dong Ren, Chen Fang, Ke Xiang, Hao-Tong Xu, Li-Jun Tang, Tao Wang, Fu-Zhou Tian

**Affiliations:** ^1^Department of General Surgery, Chengdu Military General Hospital, No. 270, Rongdu Avenue, Jinniu District, Chengdu, Sichuan 610020, China; ^2^Chengdu Military Institute for Drug and Instrument Control, No. 44 Yuefu Street, Chengdu, Sichuan 610020, China

## Abstract

*Purpose.* This study aimed to investigate the therapeutic potential of hydrogen-rich saline on pancreatic ischemia/reperfusion (I/R) injury in rats. *Methods.* Eighty heterotopic pancreas transplantations (HPT) were performed in syngenic rats. The receptors were randomized blindly into the following three groups: the HPT group and two groups that underwent transplantation and administration of hydrogen-rich saline (HS, >0.6 mM, 6 mL/kg) or normal saline (NS, 6 mL/kg) via the tail vein at the beginning of reperfusion (HPT + HS group, HPT + NS group). Samples from the pancreas and blood were taken at 12 hours after reperfusion. The protective effects of hydrogen-rich saline against I/R injury were evaluated by determining the changes in histopathology and measuring serological parameters, oxidative stress-associated molecules, and proinflammatory cytokines. *Results.* Administration of hydrogen-rich saline produced notable protection against pancreatic I/R injury in rats. Histopathological improvements and recovery of impaired pancreatic function were observed. In addition, TNF-*α*, IL-1*β*, and IL-6 were reduced markedly in the HPT + HS group. Additionally, there were noticeable inhibitory effects on the pancreatic malondialdehyde level and considerable recruitment of SOD and GPx, which are antioxidants. *Conclusion.* Hydrogen-rich saline treatment significantly attenuated the severity of pancreatic I/R injury in rats, possibly by reducing oxidative stress and inflammation.

## 1. Introduction

In recent years, pancreas transplantation has become the treatment of choice for patients with insulin-dependent diabetes mellitus [1–3]. However, graft pancreatitis as a result of ischemia/reperfusion (I/R) injury is one of the most severe complications in the early postoperative period [4]. Postimplantation pancreatitis induced by I/R is responsible for considerable morbidity after pancreas transplantation and is observed in more than 20% of the patients who undergo pancreas transplantation [5]. In cases of graft in other tissues, oxygen free radicals cause direct membrane damage, expression of adhesion molecules, and neutrophil infiltration, which mediate the damage. Many factors lead to the generation of oxygen-derived free radicals during the reperfusion period, including endothelial dysfunction, endogenous enzymes, and leucocyte recruitment [6]. The induction of oxidative stress is the major mechanism of I/R injury in pancreas transplantation [7]. Free radicals and proinflammatory cytokines could damage the cellular membrane and subcellular structures, which contain large amounts of phospholipids and protein, resulting in lipid peroxidation and subsequent structural and metabolic alterations, leading to cell apoptosis and necrosis. An early study showed that superoxide dismutase (SOD) and glutathione peroxidase (GPx) reduce the glandular edema and tissue damage induced by I/R in the pancreas [8]. Postimplantation pancreatitis induced by I/R might be caused or promoted by a prooxidative and an antioxidative imbalance. This theory led to the development of a new therapeutic strategy for reducing I/R injury based on supplementation with exogenous antioxidants.

Recently, Ohsawa and his colleagues first reported that hydrogen gas, as an antioxidant, has potential preventive and therapeutic roles in many common diseases caused by oxidative stress [9]. In 2010, Ji et al. reported that inhalation of hydrogen markedly suppressed I/R-induced brain injury by selectively eliminating toxic oxygen radicals [10]. Following that there were more studies that investigated the possible therapeutic effects of hydrogen-rich saline on renal, myocardial, intestinal, and lung I/R injuries in rats [11–14] and demonstrated that ROS and reactive nitrogen species increase in areas of ischemia and reperfusion, which are responsible for the tissue damage.

The potential effect of hydrogen on pancreatic I/R injury has not been examined. To further investigate the potential protective effect of hydrogen, we developed a rat model of heterotopic pancreas transplantation that allowed simultaneous determination of the functional, biochemical, and morphological parameters. The first aim of this study was to investigate the therapeutic effects of hydrogen-rich saline on the I/R injury of the graft after pancreas transplantation. Second, we aimed to determine the association between the protective effects of hydrogen-rich saline and its suppression of oxidative stress.

## 2. Materials and Methods

### 2.1. Animals

Male Wistar rats were purchased from the Experimental Animal Center of Sichuan University (Chengdu, Sichuan Province, China). The average weight of the rats at the time of the experiment was 200 ± 50 g. The animals were housed at a controlled temperature (22 ± 1°C) with 12-hour light and 12-hour dark cycles (light, 08:00–20:00; dark, 20:00–08:00). The animals were acclimatized for one week prior to any experimentation. The animals used in this work received humane care in compliance with institutional animal care guidelines and with approval by a committee of Sichuan University. The surgical and experimental procedures were in accordance with the institutional animal care guidelines.

### 2.2. Preparation of the Hydrogen-Rich Saline

Hydrogen-rich saline was generously provided by Professor Xue-jun Sun (at the Second Military Medical University, Shanghai, China). The saturated hydrogen-rich saline was prepared as the method described previously and stored under atmospheric pressure at 4°C in an aluminum bag [13].

### 2.3. Experimental Design

Thirty-six animals were divided into 18 donors and 18 receptors, and 18 heterotopic pancreas transplantations (HPT) were performed, with a cold ischemia time (University of Wisconsin solution at 4°C) of 12 hours. The receptors were randomized blindly into three groups and treated as follows: the HPT group, the HPT + hydrogen-rich saline group (HPT + HS), and the HPT + normal saline group (HPT + NS). The saline-treated groups received hydrogen-rich saline (>0.6 mM, 6 mL/kg) or normal saline (6 mL/kg), respectively, via the tail vein at the beginning of reperfusion. The two kinds of saline are only administered to the recipients at the time of graft reperfusion and not used in the preservation solution of the pancreas during the retrieval and cold ischemia. These animals were sacrificed at 12 hours after injury to collect the samples. Six rats that underwent only laparotomy were in the sham operation group (SO). The animals in each group were sacrificed at 12 hours to collect samples after reperfusion.

### 2.4. Pancreas Transplantation

Anesthesia: before surgery, the rats were anesthetized by inhalation of ethyl ether and an intraperitoneal injection of pentobarbital (25 mg/kg). Donor surgery: the no-touch technique was used to dislodge the pancreas, and this technique was described previously by Lee et al. [15] and modified in another study [16]. A laparotomy was performed after successful anesthesia and then the left gastric artery, hepatic artery, and common bile duct were ligated and cut. We separated the portal vein from the porta hepatis and separated the omentum from the pancreas. The pylorus and duodenum were ligated and separated, and the colon was expanded after the ligation of the colon artery and vein. We ligated the superior mesenteric artery from the head of the pancreas and separated and ligated the abdominal aorta and renal arteries. A cannula was inserted into the distal aorta, and we used 50 IU/kg of heparin to ensure anticoagulation in the animals. Thereafter, the pancreas was perfused via the aorta with cold Ringer's solution (50 mL, perfusion pressure of 50 mm Hg), and the pancreas and the portal vein containing the aortic segment were removed. Each pancreas was stored in a UW solution at 4°C for 12 hours. Recipient surgery: after the recipient laparotomy, the infrarenal aorta and vena cava were exposed and clamped with vascular clamps above the vessel bifurcation and below the level of the renal vessels. After the aorta and vena cava were incised, we made an end-to-side anastomosis by 8-0 sutures between the organ donor portal vein and inferior vena cava and between the two aorta segments. Then the saline-treated groups received hydrogen-rich saline (>0.6 mM, 6 mL/kg) or normal saline (6 mL/kg), respectively, via the tail vein at the beginning of graft reperfusion. Then, the anastomosis clamps were removed to let the donor organ reperfuse. After these procedures, an enterovesical anastomosis was performed for pancreatic secretion drainage.

The animals in each group were sacrificed at the assigned time after reperfusion to collect the samples for the biochemical assays. We reanesthetized the animals and opened the abdomen, and the serum samples were obtained from the harvested blood samples by centrifugation (1800 ×g for 15 min at 4°C). The pancreas was carefully removed, and the pancreatic tissues were removed for histopathological examination. A portion of the excised tissue was fixed in 10% neutral formalin and embedded in paraffin, and the remaining sample was immediately submerged in ice-cold NS and homogenized.

### 2.5. Histological Examination

The paraffin sections of pancreatic tissue were stained with hematoxylin-eosin after removing paraffin. Under optical microscope, the histological evaluation was performed by independent pathologists who were blind with the animal grouping. The expert graded the severity of the injuries based on edema, inflammation, and necrosis ranging from 0 to 3, as described by Schmidt et al. [17].

### 2.6. Determination of Serological Parameters Related to Pancreatic Function

The serological concentrations of insulin, lipase, and amylase were measured to assess the function of the pancreas. Insulin was measured by a radioimmunoassay using a commercial assay (INSULIN-CT; CIS Bio International, Oris Group, Gif/Yvette, France). The automated enzyme-based colorimetric assay (Hitachi 7170 biochemistry analyzer, Tokyo, Japan) was performed to determine the serum levels of amylase and lipase.

### 2.7. Determination of Serum Cytokine Levels

The serum sample was prepared for detecting the levels of tumor necrosis factor-alpha (TNF-*α*), interleukin-1*β* (IL-1*β*), and interleukin-6 (IL-6) using commercial quantitative enzyme-linked immunosorbent assay (ELISA) kits (R&D Systems, Minneapolis, MN, USA) according to the manufacturer's instructions. The protein content in the serum sample was determined by a Coomassie blue assay.

### 2.8. Detection of the Level of Malondialdehyde (MDA) and the Activities of SOD and GPx in Pancreatic Tissue

Fresh pancreatic tissue samples were placed into centrifuge tubes. Butylated hydroxytoluene was added to the homogenized pancreatic tissues with protease inhibitors. Then, the mixture was centrifuged at 10,000 ×g for 10 min at 4°C. The supernatant was used for the determination. The level of MDA in the pancreatic tissue was detected using a commercial MDA-586 assay kit (OxisResearch, Portland, OR, USA) according to the manufacturer's instructions. The MDA levels in the tissue were normalized against the total protein (pmol/mg). The SOD activity in the pancreas was measured using a commercial assay kit (Ann Arbor, MI, USA), following the manufacturer's instructions. This assay kit utilizes tetrazolium salt for the detection of superoxide anions generated by xanthine oxidase and hypoxanthine. The total tissue protein concentration was determined using a commercial kit (Nanjing Jiancheng Corp., Nanjing, China), and the SOD activity is expressed as U/mg of protein. The GPx activity in the pancreatic tissue was measured according to the method described by Paglia and Valentine [18]. GPx catalyzes the oxidation of glutathione (GSH) by H_2_O_2_, and nicotinamide adenine dinucleotide phosphate (NADPH) (reduced form) is converted to NADPH+ (oxidized form) during the reaction; GPx activity can thus be detected by the absorbance at 340 nm.

### 2.9. Statistical Analysis

All of the experimental results are presented as the mean ± standard deviation (SD). Significant differences in the data between groups were compared by Student's *t*-test. A *P* value of less than 0.05 was considered statistically significant.

## 3. Results

### 3.1. Histopathologic Changes

**Figure 1:**
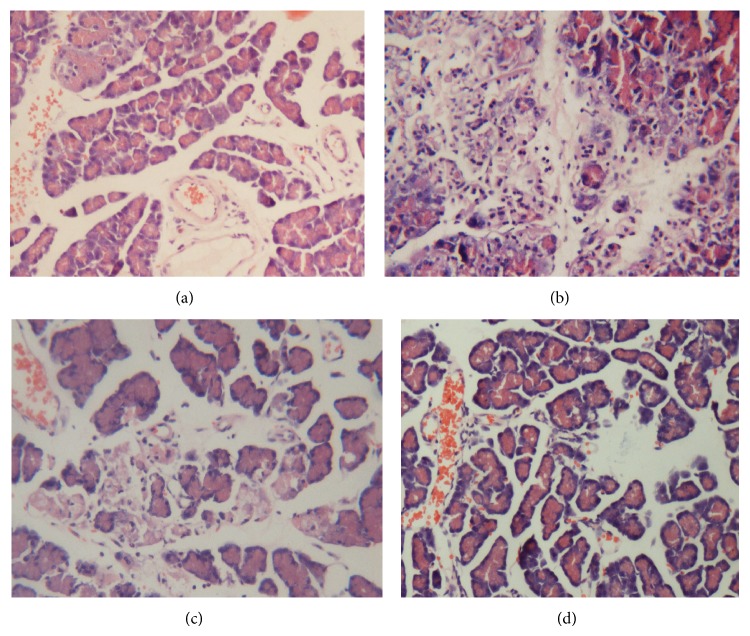
Histopathologic analysis of the pancreas using hematoxylin-eosin staining (×200). (a) SO group: pancreatic acini are well conserved without edema, inflammatory infiltrates, or hemorrhage. ((b), (c)) HPT and HPT + NS groups: acute pancreatitis characterized by interacinar edema, inflammatory infiltrates, necrosis, and hemorrhage. (d) HPT + HS group: these histological changes showed significantly less extensive inflammatory infiltrates, interacinar edema, necrosis, and hemorrhage. Bar equals 50 *μ*m.

Histopathological examination of the pancreas demonstrated that all of the animals undergoing transplantation showed signs of a postischemia pancreatitis with significant edema formation, inflammatory infiltration, and cell necrosis. In the HPT + HS group, these histological changes were significantly less evident compared with the animals that received normal saline before reperfusion, as evidenced by the histopathological scoring (Table 1) (Figure 1). These results showed that the hydrogen-rich saline might relate to the relieving of histopathological changes of pancreatic tissue.

### 3.2. Serological Parameters Related to Pancreatic Function

**Figure 2:**
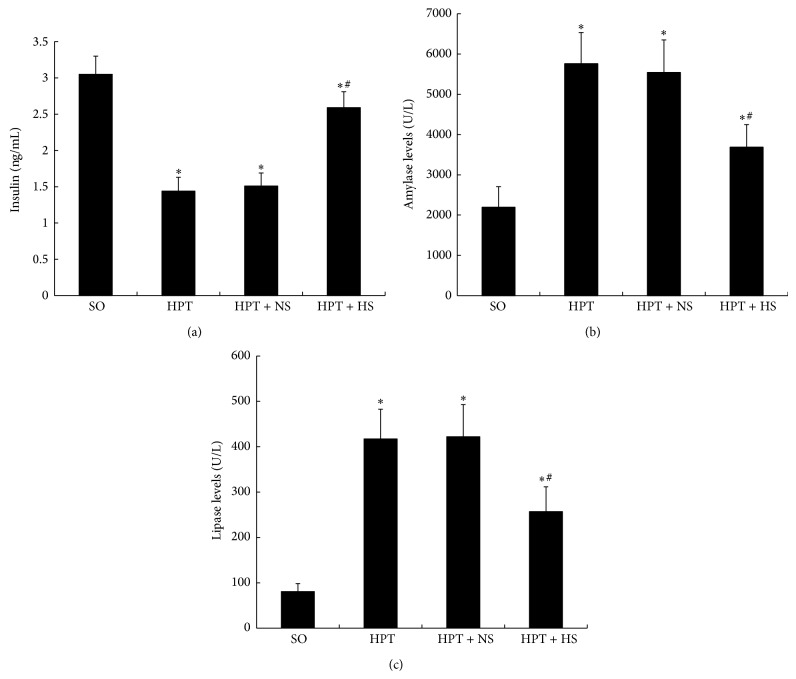
Changes in insulin (a), amylase (b), and lipase (c) serum concentrations in rats exposed to various treatments. As with the results of oxidative stress after I/R injury, the levels of insulin were decreased, whereas the levels of amylase and lipase were increased. The treatment with hydrogen-rich saline reverted those parameters to basal levels. ^*^ *P* < 0.05 compared with the SO group; ^#^ *P* < 0.05 compared with the HPT + NS group.

I/R induced a significant reduction in the insulin concentration in the serum (*P* < 0.05) (Figure 2(a)). The administration of hydrogen-rich saline partially restored the level of insulin in the HPT + HS group and caused a noticeable increase compared with the HPT + NS group. There were marked increases in the amylase and lipase levels in the serum of rats subjected to I/R injury in the HPT group, HPT + NS group, and HPT + HS group compared with the SO group (*P* < 0.05) (Figures 2(b) and 2(c)). Hydrogen-rich saline administration significantly reduced the amylase and lipase levels in the HPT + HS group compared with those in the HPT + NS group (*P* < 0.05). These results indicated that the hydrogen-rich saline might be helpful for the recovery of endocrine function of pancreas and it reduced the severity of graft pancreatitis.

### 3.3. Serum Cytokine Levels

**Figure 3:**
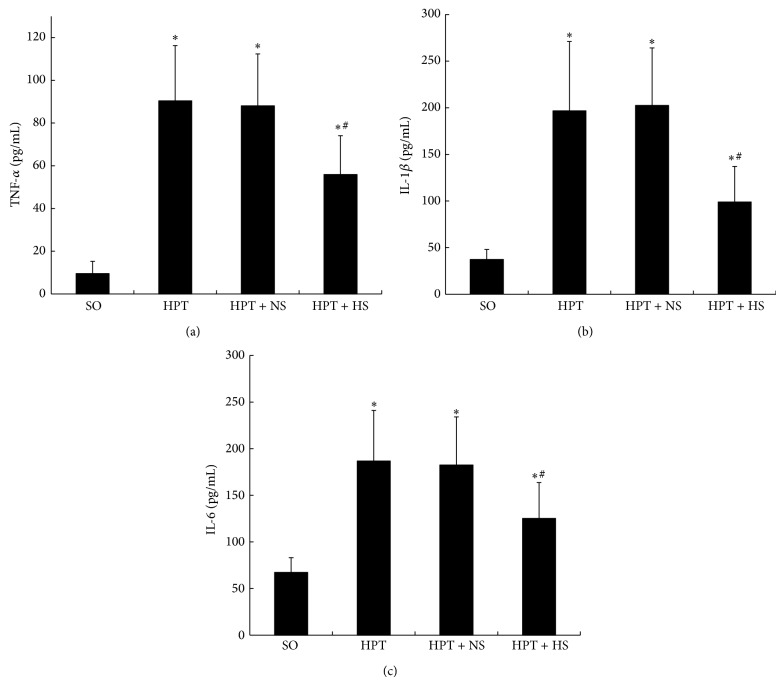
Inhibition of hydrogen-rich saline treatment on serum cytokine levels in rats. The serum TNF-*α* (a), IL-1*β* (b), and IL-6 (c) levels increased strongly after I/R injury. The administration of hydrogen-rich saline (0.6 mM, 6 mL/kg) resulted in significantly decreased levels of serum TNF-*α*, IL-1*β*, and IL-6. ^*^ *P* < 0.05 compared with the SO group; ^#^ *P* < 0.05, compared with the HPT + NS group.

Compared with the SO group, the levels of serum TNF-*α*, IL-1*β*, and IL-6 were remarkably increased in the HPT group, HPT + NS group, and HPT + HS group (*P* < 0.05). In addition, treatment with hydrogen-rich saline partially reversed those parameters of serum cytokines; the levels were significantly lower than the levels after treatment with normal saline (Figure 3). Our results reveal that treatment with hydrogen-rich saline attenuates I/R injury-induced inflammation in the pancreas.

### 3.4. Oxidative Stress-Associated Molecules

**Figure 4:**
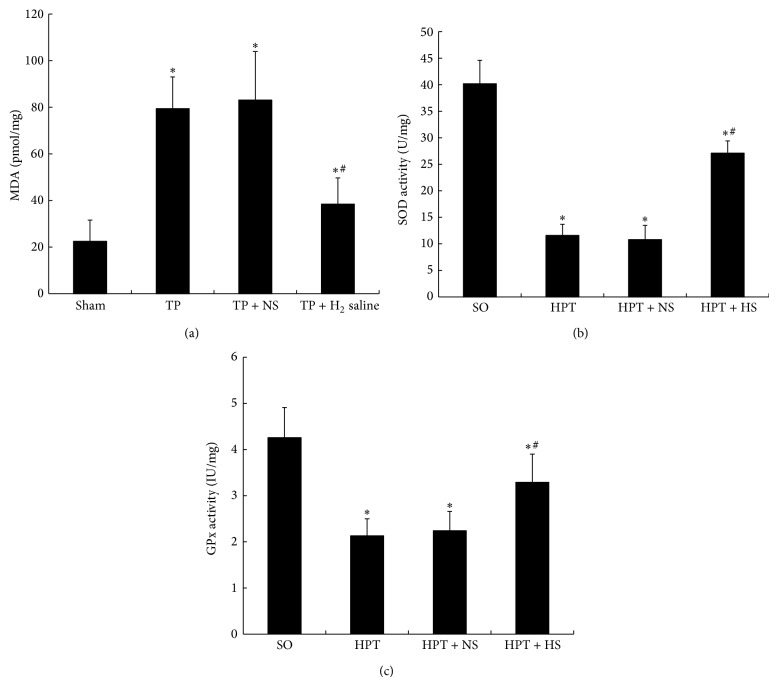
Pancreatic tissue levels of MDA, SOD, and GPx in rats exposed to various treatments. After I/R injury, the MDA level (a) in pancreatic tissue presented a pronounced increase, and the SOD (b) and GPx (c) in the pancreas were nearly depleted. The administration of hydrogen-rich saline (0.6 mM, 6 mL/kg) effectively improved the SOD and GPx levels in the pancreas with significantly reduced pancreatic MDA levels. ^*^ *P* < 0.05 compared with the SO group; ^#^ *P* < 0.05 compared with the HPT + NS group.

As a marker of lipid peroxidation, pancreatic MDA levels indicate the degree of oxidative stress caused by I/R injury. Although enhanced MDA levels were observed in all of the injured animals, the elevation appeared to be significantly inhibited by hydrogen-rich saline treatment (*P* < 0.05; Figure 4(a)). Moreover, as a result of oxidative stress-induced processes, the SOD activity of pancreatic tissue in rats suffering from I/R injury was markedly reduced compared with the sham operation group. Treatment with hydrogen-rich saline significantly attenuated this reduction (*P* < 0.05; Figure 4(b)). Furthermore, pancreatic GPx activity was notably decreased in injured rats; however, treatment with hydrogen-rich saline effectively improved the activity of GPx in the pancreas (*P* < 0.05; Figure 4(c)). Our results revealed that treatment with hydrogen-rich saline attenuates I/R injury-induced oxidative stress in the pancreas.

## 4. Discussion

In the present study, I/R induced intense oxidative stress, which was characterized by an increase in lipid peroxides and the depletion of enzymatic (SOD and GSH-Px) antioxidants, in the pancreas. Treatment with hydrogen-rich saline exerted a powerful antioxidant effect with a reduction in free radical-derived product MDA and recovery of antioxidant status in the pancreas subjected to I/R. This finding suggests that hydrogen might exert its therapeutic effects on I/R injury by inhibiting the excessive activation of oxidative stress or by correcting the prooxidative/antioxidative imbalance.

I/R injuries are issues which are relevant for all organ transplantations [19] that are clearly worsened in individuals with diabetes due to the high levels of oxidative damage [20]. I/R induces the development of postimplantation pancreatitis, which is responsible for considerable morbidity following pancreas transplants. The graft pancreatitis induced by I/R might involve various factors, thereby stimulating different molecular pathways and leading to different physiological complications. The role of oxidative stress in the I/R injury of the pancreas is well demonstrated. Oxygen free radicals induce cell and tissue damage not only by causing direct cellular membrane damage but also by enhancing the expression of adhesion molecules and promoting inflammatory infiltration.

Inflammatory infiltration plays an important role in the development of pancreatic damage during the course of I/R injury. Cytokines such as TNF-*α*, IL-1*β*, and IL-6 play important roles in the induction of neutrophil activation and infiltration and induce localized tissue injury and remote organ injury [21]. In the present study, the pancreas tissue levels of the cytokines TNF-*α*, IL-1*β*, and IL-6 were significantly elevated in I/R-induced pancreas injury. Lipid peroxidation caused by ROS is one of the most critical mechanisms leading to cellular damage. Hydroxyl radicals attack membrane-associated polyunsaturated fatty acids, leading to lipid peroxidation and cellular damage [22]. MDA is a sensitive indicator of oxidative DNA damage. SOD converts the superoxide anion radical into H_2_O_2_, which is detoxified into H_2_O by antioxidant enzymes such as GPx. Therefore, deficiencies of these antioxidant enzymes could cause excessive oxidative stress. Our study demonstrated that hydrogen-rich saline treatment significantly alleviated oxidative stress following I/R injury by reducing the MDA levels and improving the activities of SOD and GPx in the pancreas.

Oxidative stress and its resultant production of ROS play key roles in the pathogenesis of pancreatitis induced by I/R injury; many researchers have been studying the therapeutic potential of antioxidants. Muñoz-Casares's study showed that the administration of melatonin as an antioxidant prevented some tissue markers of oxidative stress [23]; another study showed that melatonin also improves interleukin-10 (IL-10), TNF-*α*, and amylase concentrations in serum during I/R injury [24]. However, the majority of antioxidants have not shown sufficient capability to protect oxidative stress-induced injury. Meanwhile, it was demonstrated that there was no evidence to justify the continued administration of antioxidant-based therapy with n-acetylcysteine, selenium, or vitamin C in severe acute pancreatitis [25]. Otherwise, treatment with antioxidants, such as beta carotene, vitamin A, and vitamin E, may lead to physiologic disorders and increase mortality in some oxidative stress-related diseases [26].

Recently, Ohsawa et al. reported that hydrogen could selectively reduce the cytotoxic oxygen radicals induced by I/R injury [9]. Furthermore, Chen et al. demonstrated that hydrogen-rich saline has the protective effects of hydrogen-rich saline on the L-arginine-induced acute pancreatitis due to its ability to inhibit oxidative stress, apoptosis, and NF-kB activation [27]. Hydrogen has the ability to rapidly diffuse across cellular membranes, react with cytotoxic ROS, and subsequently protect against oxidative damage. Thus, hydrogen is considered as an established, safe, and convenient antioxidant [9]. This property of hydrogen provides valuable insights into the potential mechanisms of ameliorating I/R injury-induced graft pancreatitis. Previous studies have indicated that the ROS such as ^*∙*^OH and oxidative stress-related mediators dramatically increased after reperfusion of pancreatic grafts with 18-hour cold ischemia preservation in rat. Consistent with these reports, the current study also showed that the oxidative stress-related mediators, such as MDA, SOD, and GPx, underwent marked increase after reperfusion during pancreatic transplantation, and administration of hydrogen-rich saline resulted in significant decrease of these mediators. So, we presumed that hydrogen-mediated clearance of ^*∙*^OH and other detrimental ROS might play an important role in relieving the oxidative stress of pancreatic I/R injury. However, beyond pancreatic transplantation, other animal models, such as pancreatic warm ischemic injury model, should be established to further investigate the role and mechanisms of hydrogen in the protection of pancreatic I/R injury.

## 5. Conclusion

Our findings indicate that hydrogen-rich saline has anti-inflammatory and antioxidant effect. It is a promising therapy for graft pancreatitis induced by I/R injury in rats. Pancreas I/R injuries, such as vascular surgery, transplantation, and pancreatic injury, frequently occur because of surgical or medical causes. Further studies are required to confirm the clinical values and reveal the detailed mechanisms of hydrogen-rich saline.

## Figures and Tables

**Table 1:** Histopathological scores of pancreatic injury.

Groups	SO (*n* = 6)	HPT (*n* = 6)	HPT + NS (*n* = 6)	HPT + HS (*n* = 6)
Edema	0	1.9 ± 0.4	2.0 ± 0.4	0.9 ± 0.2^*^
Inflammation	0	2.0 ± 0.2	2.1 ± 0.3	1.0 ± 0.1^*^
Necrosis	0	1.8 ± 0.3	1.8 ± 0.3	1.0 ± 0.2^*^
Histopathological scores	0	5.7 ± 0.8	5.9 ± 0.7	2.9 ± 0.4^*^

The data are expressed as the mean ± SE. ^*^ *P* < 0.05 versus the HPT + NS group.
